# Radiation-induced abscopal reproductive effect is driven by TNF-α/p38 MAPK/Rac1 axis in Sertoli cells

**DOI:** 10.7150/thno.56853

**Published:** 2021-03-31

**Authors:** Songling Hu, Lin Zhu, Yimeng Song, Xinrui Zhao, Qianping Chen, Yan Pan, Jianghong Zhang, Yang Bai, Haowen Zhang, Chunlin Shao

**Affiliations:** 1 Institute of Radiation Medicine, Shanghai Medical College, Fudan University, Shanghai 200032, China.; 2 State Key Laboratory of Radiation Medicine and Protection, School of Radiation Medicine and Protection, Medical College of Soochow University, Suzhou 215123, China.

**Keywords:** thoracic irradiation, abscopal effect, testis damage, rac1 nuclear translocation, TNF-α/p38 MAPK pathway

## Abstract

**Rationale:** Radiotherapy has become a mainstay for tumor management, and more than 50% of patients with thoracic tumor need to be treated with radiotherapy. However, the potential adverse effects of thoracic radiotherapy on the reproductive system remain elusive.

**Methods:** Western blot analysis, immunofluorescence assay and transmission electron microscopy (TEM) analysis were performed to investigate the integrity of blood-testis barrier (BTB) in male mice after hypofractionated irradiation (IR) on the right thorax. RNA sequencing, co-immunoprecipitation (IP), Duolink PLA and inhibitor experiments were carried out to demonstrate the molecular mechanisms of the BTB dynamics changes and the subsequent reproductive effect.

**Results:** It was found that the hypofractionated IR on right thorax evoked ultrastructural destruction in distant testes, and thus caused radiation-induced abscopal reproductive effect (RIARE) in male mice. Mechanistically, thoracic IR induced significant nuclear translocation of Rac Family Small GTPase 1 (Rac1) in abscopal Sertoli cells, which closely correlated with the activation of TNF-α/p38 mitogen activated protein kinase (MAPK) pathway. Of note, YWHAZ, a critical polarity protein, was found to be co-localized with Rac1 in Sertoli cells, and this interaction was indispensable for thoracic IR-induced Rac1 nuclear translocation and subsequent degradation of BTB-associated proteins.

**Conclusions:** Our findings imply for the first time that YWHAZ-mediated Rac1 nuclear translocation plays central roles in RIARE, and TNF-α/p38 MAPK/Rac1 axis can be employed as a therapeutic target against RIARE for young male patients receiving hypofractionated radiotherapy.

## Introduction

Radiotherapy, a prominent frontline therapy, generally contributes more than a half to curing cancers, which is most effective when compared with other treatments such as traditional chemotherapy and molecular-targeted treatment (excluding surgery) 1. Recently, immunotherapy has radically changed the clinical treatment of tumor patients, accompanied by tremendous advances in cancer immunology, and hypofractionated radiotherapy has become promising strategies for clinical tumor immunotherapy as it sensitizes refractory tumors to immune checkpoint inhibitors by inducing cancer cell death coupled with danger signal release and recruitment of anti-tumor T cells 2-6. However, hypofractionated radiotherapy may also cause severe toxicity in normal tissues such as treatment-related pulmonary toxicities 7 or enterocolitis 8. The growing number of male cancer survivors of childbearing age and the increasing awareness of the potential mutagenic effects of radiochemotherapy has raised great concerns about the health of tumor patient's offspring. Danish and Swedish registries including about 1.78 million singleton children born between 1994 and 2004 (Denmark) and 2005 (Sweden) have provided a statistically significant increase in the risk of major congenital abnormalities among offspring of male cancer survivors 9. Radiation-induced abscopal effect in testes was first described in 2008, Jan *et al*. confirmed that localized paternal cranial irradiation (IR) could induce DNA damage in distant sperm cells and lead to profound epigenetic dysregulation in the unexposed rat offspring 10. However, whether IR could induce observable non-target reproductive effect in male mice based on impaired spermatogenesis remains controversial.

Spermatogenesis in the seminiferous epithelium is safeguarded by a specific cellular ultrastructure that is composed of testicular Sertoli cells. The Sertoli cell seminiferous epithelium barrier, also known as the blood-testis barrier (BTB), is constituted mainly by tight junctions (TJs) along with the co-existing of basal ectoplasmic specialization (ES), desmosome and gap junctions. The basal ES is a unique F-actin cytoskeletal structure that ensures the proper attachment of developing spermatogenic cells to Sertoli cells 11. Besides, the TJs physically divide the seminiferous epithelium into basal and apical compartments, which prevents the entry of deleterious endogenous or exogenous substrates, thereby providing a stable environment for spermatogenesis 11, 12. Nonetheless, lots of environmental contaminants can induce BTB disruption and fertility disorders. For example, CdCl_2_ can induce loss of BTB-associated proteins 13 such as occludin and zonula occludens-1 (ZO-1) from the TJs in two adjacent Sertoli cells by phosphorylating p38 mitogen activated protein kinase (MAPK) 14. The p38 MAPK signaling pathway is a critical downstream target of tumor necrosis factor-α (TNF-α) and transforming growth factor-β (TGF-β) and is activated in the process of BTB reconstruction during spermatogenesis 15, 16. However, the role of p38 MAPK in radiation-induced abscopal reproductive effect (RIARE) remains elusive.

The Rho GTPase family has been demonstrated to be one of the downstream targets of p38 MAPK pathway 16, which plays a broad range of roles in cell movement, cell adhesion, regulation of cytoskeleton, even cell death and survival 17. The Rho GTPases are small GTP-binding proteins belonging to the Ras superfamily, within which the Rac subfamily has been most extensively characterized 18. The most well-known member of Rho GTPase family is Rac Family Small GTPase 1 (Rac1) that has a diverse repertoire of cellular functions. However, little is known about the specific function of Rac1 in Sertoli cells and spermatogenic cells. Recently, it has been proposed that Rac1 acts as a downstream of mechanistic target of rapamycin kinase complex 1 (mTORC1) to establish the cytoarchitecture of Sertoli cells 19. Although the function of Sertoli Rac1 is dispensable for fetal testicular differentiation and the maintenance of undifferentiated spermatogonia, in the adult testis, Rac1 deficiency results in severe defects in cell polarity and disruption of the BTB 20. Sertoli cell polarity is mainly regulated by proteins such as PAR6 and YWHAZ (also known as 14-3-3ζ), and confers cell adhesion at the interface of adjacent Sertoli cells on BTB 21. Both YWHAZ family and Rho GTPases could regulate cell cytoskeleton remodeling, suggesting a potential mutual regulation of these two signaling pathways 22. Nevertheless, the specific roles of Sertoli cell polarity in RIARE are not defined. Moreover, the molecular patterns of protein-protein interactions between polarity proteins and BTB-associated proteins are still not fully understood.

In the present study, we find that the hypofractionated irradiation (HF-IR) of the right thorax of mice induces abscopal reproductive effects, which is driven by apparent Sertoli Rac1 nuclear translocation and degradation of BTB-associated proteins. Importantly, the interaction of polarity protein of YWHAZ with Rac1 is indispensable for thoracic IR-induced Rac1 nuclear transport in Sertoli cells. These findings suggest that the subcellular localization of Sertoli Rac1 plays central roles in RIARE, and targeting Rac1 may be promising in attenuating harmful RIARE in young male patients receiving hypofractionated radiotherapy.

## Materials and Methods

### Animals and cell lines

Mature male C57BL/6 mice were purchased from Shanghai Model Organisms Center, Inc. (Shanghai, China). If not otherwise stated, only male mice were randomly assorted and blindly assigned to experimental groups. All experimental animals were conducted 7-8 weeks of age. The study was conducted in compliance with local animal welfare laws, guidelines, and policies. All procedures were approved by the ethic committee of Soochow University (Approval No. ECSU-2019000150).

TM4 Sertoli cell lines were purchased from ATCC and were culture in DMEM/F12 medium supplemented with 5% horse serum and 5% fetal bovine serum and maintained at 37 ºC in a humidified atmosphere with 5% CO_2_.

### Mouse irradiation and mouse mating

Mice were divided into 3 groups with an equal number of 12 per group. Group I, received normal condition except radiation (abbreviated as “Control”). Group II, maintained the same procedure in the irradiating apparatus except radiation (abbreviated as “Sham IR”). Group III, the IR group which were locally irradiated through right thorax in a dimension of 1×1 cm^2^ by hypofractionated dose of X-rays (8 Gy at a time in consecutive 3 days, 24 Gy in total) at a dose rate of 0.883 Gy/min (X-RAD 320, PXI Inc., North Branford, CT, USA; 12 mA, 2-mm aluminum filtration). Before IR, the mice were anesthetized with ketamine.

The mating experiments were conducted independently for 3 times to evaluate the fertility of above irradiated male mice lasted for 42 days after IR based on the spermatogenic cell cycle 23, 24. After HF-IR (8 Gy × 3), virgin male mice were mated with non-irradiated virgin female mice at 1, 2, 3, 4, 5, 6 week after IR, and the pregnancy rate of females was counted.

### Sperm concentration and sperm vitality

Mice were divided into 3 groups (Control, Sham IR, and 8 Gy × 3) with an equal number of 8 per group. At different time after HF-IR, the epididymal caudas were isolated from testis, and the connective tissue and blood vessels were carefully removed. Sperm suspensions were prepared by mincing epididymal caudas in PBS. The suspension was pipetted and filtered through 70 μm cell strainer (BD Falcon, Cat. 352350) to remove tissue fragments. Epididymal sperm suspension was used to determine the sperm concentration using a blood counting chamber under an optical microscope (Olympus, Japan). Using a MTT kit, the sperm vitality was measured by a microplate reader (Infinite M200Pro, Tecan, Switzerland).

### RNA sequencing and data analysis

Mice were divided into 2 groups (Sham IR and 8 Gy × 3) with an equal number of 3 per group. Mouse testis tissues (3 testes/group) were extracted on the 3rd day after irradiation and frozen at -80 ºC. And then the testis tissues were sent to Shanghai Genomic Institution (BGI, Shenzhen, China) for RNA extraction, cDNA library construction and sequencing. Samples were performed on BGISEQ-500RS platform. The average alignment ratio of the sample comparison genome was 94.21%. Raw sequencing data are available at Sequence Read Archive (SRA) database and connected to bioproject PRJNA510858.2.

Genes with a fold change (FC) greater than 1.2 with a p-value less than 0.05 were considered as differentially expressed genes (DEGs). The functional enrichment analysis of DEGs was generated based on KEGG database.

### Drug treatment

Specific inhibitor of p38 MAPK, SB202190 (10 μM, S7067, Sigma) and SB203580 (10 μM, T1764, TargetMol), and a highly selective TNF-α secretion inhibitor lenalidomide (CC-5013) (10 μM, S1029, Selleckchem) were all dissolved in dimethyl sulfoxide (DMSO), subsequently diluted in 0.1 M phosphate-buffered physiological saline (PBS) and sterilized through a 0.22-µm filter. For *in vivo* study, SB202190 (25 μg/kg/d), SB203580 (50 mg/kg/d) or lenalidomide (50 mg/kg/d) was pretreated intraperitoneally once a day on 3 consecutive days and 2 h ahead of HF-IR as well. SB202190 and SB203580 was injected respectively, and 0.1% DMSO was used as solvent control.

In addition, recombinant murine TNF-α/TNFSF2 (P6020, Beyotime, China) at a working concentration of 1.0 ng/ml was administrated to TM4 Sertoli cells *in vitro* for different times.

### Assessment of spermatogenesis defects by HE

Defects in spermatogenesis were assessed based on the histology of the cross-sections of paraffin-embedded testes 25. After euthanasia of mice, the specimens of testis were fixed in Bouin's solution for 24 h before embedding in paraffin. The sections in 5 μm thickness were stained with hematoxylin and eosin (HE), and their histological structures were observed. Images of testis sections were acquired using Nikon Eclipse E100 microscope and processed with NIKON DS-U3 software package without any further manipulations. All histological data reported herein were representative findings from experiments of 8 mice/group including Sham IR and thoracic IR groups.

### Primary Sertoli cell isolation

Primary Sertoli cells were isolated from 7-day-old C57BL/6 mice using the standard enzymic method described previously 26. After 24 h culture, the Sertoli cells were subjected to hypotonic solution (20 mM Tris, pH 7.4) for 3 min at room temperature to purify Sertoli cells. As such, these cultures had a Sertoli cell purity of approximately 98%, with negligible germ and Leydig cell contamination.

### Western blot assay

Proteins of either testes or TM4 Sertoli cells were extracted, resolved via SDS-PAGE, and were electrophoresed on 7.5 or 10% polyacrylamide gel using an electrophoresis system (Bio-Rad Laboratories Inc., CA, United States), then transferred onto polyvinylidene fluoride membranes (Millipore, Cat. ISEQ00010). The membranes were blocked with 5% skim milk and then incubated overnight with primary antibodies at 4 °C. Antibodies used in the experiments: Rac1 (ab33186, Abcam), YWHAZ (ab51129, Abcam), TNF-α (ab183218, Abcam), ZO-1 (ab216880, Abcam), Occludin (33-1500, Invitrogen), F-actin (ab130935, Abcam), p38 (ab170099, Abcam), p-p38 (ab195049, Abcam), β-actin (AF0003, Beyotime), α-tubulin (AF0001, Beyotime), Lamin A/C (A19524, Abclonal). After incubation with HRP-conjugated secondary antibodies, protein bands were detected and visualized by the enhanced chemiluminescence system (ECL kit, Millipore, St. Louis, MO, United States), and band images were analyzed with the Bio-Rad ChemiDoc XRS system.

### Immunofluorescence staining

Immunofluorescence staining (include dual-labeled co-localization) was performed as previous described 25, 27, 28. In brief, the cross-sections (7 μm thick) of testis were fixed in 4% paraformaldehyde (PFA), permeabilized with 0.1% Triton X-100, non-specific binding sites were blocked in 1% BSA (wt/vol) in PBS and then incubated overnight with corresponding primary antibodies (anti-ZO-1, anti-Occludin, anti-Rac1, anti-α-tubulin, anti-YWHAZ) at a dilution of 1:50.

To show the binding between Rac1 and YWHAZ, the cross-sections of testis were subjected to Duolink proximity ligation assay (PLA) *in situ* (DUO92101, Sigma-Aldrich) staining with anti-Rac1 (ab33186, Abcam) and anti-YWHAZ (ab51129, Abcam) primary antibodies according to the manufacturer's instruction as previously described 29. The images were captured using a confocal microscope (Leica SP8, Wetzlar, Germany).

TM4 Sertoli cells grown on coverslips were fixed by 4 % PFA, penetrated, and stained by anti-F-actin antibody (1:50, ab130935, Abcam) at 4 °C overnight. Cells were incubated with the secondary antibodies anti-IgG rabbit Alexa Alexa Fluor®488 (Thermo Fisher Scientific, USA) at 1:800 in the dark for 1 h, and co-stained with 4′,6-diamidino-2-phenylindole (DAPI, Beyotime Biotechnology, Haimen, China) to visualize cell nuclei.

### RNA interfering

Verified Rac1 and YWHAZ siRNA sequences (GenePharma Co. Ltd., Shanghai, China) were applied as follow: GCATTTCCTGGAGAGTACA (Rac1) and CCATTGCTGAACTTGATACAT (YWHAZ). TM4 Sertoli cells were seeded onto six-well plates (1×10^5^/well), and then transiently transfected with 20 nM of Rac1 siRNA (siRac1), YWHAZ siRNA (siYWHAZ) or scramble siRNA using Lipofectamine 3000 and Opti-MEM® (Gibco Invitrogen) according to the manufacturer's protocol. After incubation for 48 h, the siRNA interfering efficiency was identified using Western blot assay.

### Generation of conditioned medium

At 1 day after HF-IR, the mouse serum from different groups was collected as the conditioned serum (CS). Then the primary Sertoli cells and TM4 cells were treated with medium containing 10% CS for different times to detect the cytotoxicity effects of CS.

### Transepithelial electrical resistance (TER) measurement

TER across cell epithelium was monitored to assess the TJ permeability barrier with a Millicell ERS system (Millipore Corp., Bedford, MA) as described previously 26. Primary Sertoli cells derived from 7-day-old mice were seeded onto Matrigel-coated insert dish (PC bottom membrane with 4.67 μm pores) in a transwell plate at a density of 3×10^6^ cells/cm^2^. When TER value reached plateau by 3 days after planting, cells were treated with medium containing 10% CS from irradiated mice. TER value was detected in each unit at four different areas (12-, 3-, 6-, and 9-o'clock positions) every 24 h, which were averaged into a single value and presented as “R”. The blank control was conducted in the unit with only DMEM/F12 medium. The true TER value of each sample was calculated as: TER_sample_ (Ω cm^2^) = (R_sample_ - R_blank_) (Ω) × Effective Membrane Area (cm^2^).

### Transmission electron microscopy (TEM) analysis

TEM was used to examine the ultrastructure integrity of BTB after HF-IR on right thorax. In brief, testes were cut into pieces to release the seminiferous tubules. Approximately 1 mm × 1 mm × 1 mm testis tissue was fixed by immersion into 2.5% glutaraldehyde then fixed in 1% osmium tetroxide, dehydrated in a graded series of ethanol. After dehydration, tubules were incubated in propylene oxide for 45 minutes, and infiltrated with EMbed (Electron Microscopy Sciences, Fort Washington, PA, USA). Sections were examined and photographed on a JEOL 100CXII electron microscope at 80 kV. Electron microscopy was performed at the Servicebio Bio-Imaging Resource Center (Shanghai, China).

### WT and MT Rac1 transfection and selection

Rac1 was cloned into the *CMV-MCS-3FLAG-SV40-Neomycin* to generate Rac1 overexpressing vector. In order to generate the MT Rac1 construct, the PVKKRKRK sequence at amino acid 181-188 of WT Rac1 was replaced with RRGKKKSG. To establish cells stably overexpressing WT-Rac1 or MT-Rac1, 48 h after transfection, the TM4 Sertoli cells were treated with Neomycin for 1 week.

### Tandem mass tag (TMT) quantitative proteomic analysis

The protein contents in the mice serum were evaluated by the liquid chromatography in combination with tandem mass spectroscopy (LC-MS/MS), which was performed by Shanghai Genechem Co., Ltd. Briefly, protein digestion and labelling with iTRAQ Reagent 8-plex Multiplex kit (Sciex) were performed according to manufacturer's instruction. After centrifugation (12,000 g, 4 °C, 10 min) to remove the remaining debris, the supernatant was collected and the protein concentration was determined with a BCA kit (Q10) according to the manufacturer's instruction. The tryptic peptides were then digested into fractions and separated by high pH reverse-phase HPLC based on Agilent 300 Extend C18 column (5 mm particles, 4.6 mm ID, 250 mm length) and subjected to a NSI source followed by tandem mass spectrometry (MS/MS) in a Q ExactiveTM Plus (Thermo, MA, United States) coupled online UPLC. The MS/MS data were processed with the Maxquant search engine (v.1.5.2.8).

### Co-immunoprecipitation (Co‑IP) assay

Co-IP was performed by using 500 μg of protein from the lysates of testes. Anti-Rac1 antibody (1 μg, ab33186, Abcam, Cambridge, MA) was used as the precipitating antibody. Anti-YWHAZ (1:1000, ab51129, Abcam, Cambridge, MA), anti-F-actin (1:1000, ab130935, Abcam, Cambridge, MA) and anti-α-tubulin (1:1000, AF0001, Beyotime, China) were used for Western blot analysis. Finally, the protein bands were visualized by ChemiDoc XRS system (Bio-Radt Laboratories Inc., Hercules, CA, USA).

### Enzyme-linked immunosorbent assay of TNF-α and TGF-β

Mice were divided with an equal number of 8 per group. The concentrations of TNF-α and TGF-β in the mice serum were measured with mouse TNF-α and TGF-β ELISA kit (Thermo Fisher Scientific, Carlsbad, CA, USA), respectively, according to the manufacturer's instruction. After reaction, the optical density (OD) at 450 nm of each sample was detected by a microplate reader (Tecan Infinite 200 Pro, Männedorf, Switzerland). Each sample was measured in triplicate.

### Statistical analysis

Data were expressed as mean ± SD from at least three independent experiments. Differences between groups with similar variance were analyzed by Student's t-test. Asterisks represent the *p* values as follows: *p < 0.05, **p < 0.01 and ***p < 0.001.

## Results

### P38 MAPK inhibitor alleviates RIARE *in vivo*

**Figure 1:**
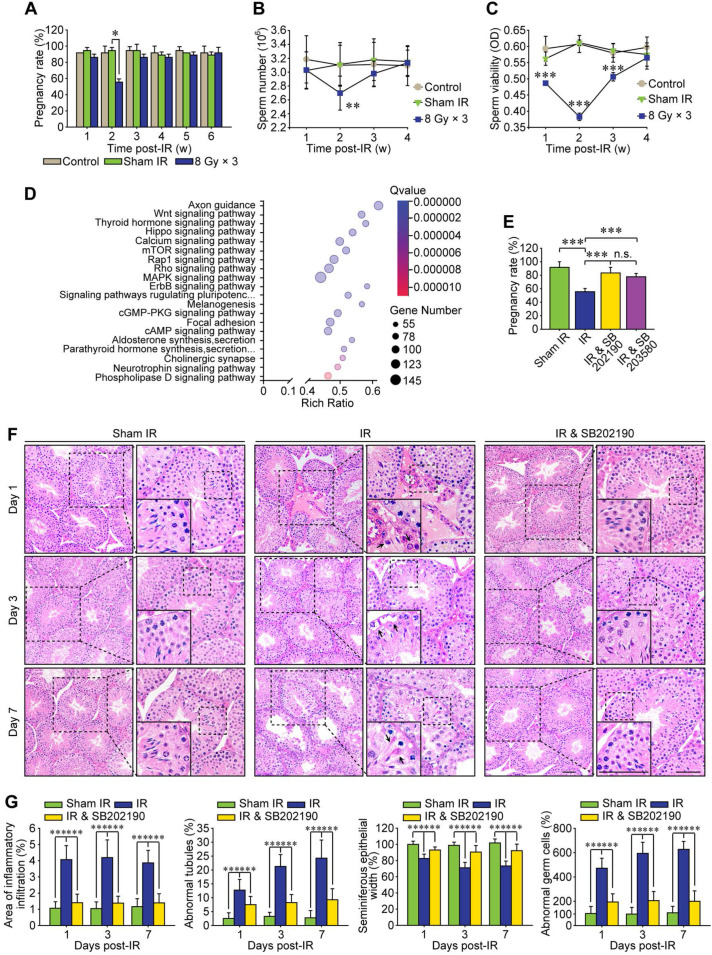
** P38 MAPK inhibitor alleviates radiation-induced abscopal reproductive effect (RIARE) *in vivo*. (A)** Thoracic-irradiated (8 Gy × 3) male mice were mated with virgin female mice at the indicated time after IR, and the pregnancy rate of female mice was evaluated (n=12). Sperm concentration **(B)** and sperm viability **(C)** in cauda epididymidis were analyzed at the indicated time after thoracic IR. **(D)** Functional enrichment analysis of RNA sequencing of abscopal testes of thoracic-irradiated male mice at 3 days post-IR. **(E)** Each thoracic-irradiated male mice (n=12) were mated with one virgin female mice at 2 weeks after IR with or without pre-treatment of SB202190 or SB203580, and the pregnancy rate of female mice was evaluated (n=12). SB202190 (25 μg/kg/d, i.p. for 3 days) or SB203580 (50 mg/kg/d, i.p. for 3 days) was administered to male mice 2 h ahead of HF-IR.** (F)** Representative images of HE staining of seminiferous tubules after thoracic IR with or without SB202190 pre-treatment (n=8). SB202190 was administered to mice (25 μg/kg/d, i.p. for 3 days) 2 h ahead of hypofractionated radiation (HF-IR) on right thorax. Scale bars = 20 µm.** (G)** The ratio of seminiferous tubules with disorganized seminiferous epithelium or giant cell formation under different conditions shown in Figure 1F. *p < 0.05, **p < 0.01, ***p < 0.001.

Increasing preclinical and clinical data indicate that both total radiation dose and its fractionation procedure influence the radioimmunotherapy efficacy 30. Compared with single 20 Gy radiotherapy, a total dose of 24 Gy given in three consecutive days (*i.e.*, 8 Gy × 3) achieves more apparent abscopal anti-tumor responses in mice 31. Therefore, a localized right thoracic radiation regimen of 8 Gy × 3 was carried out in the present study to disclose the non-target adverse effects in male reproduction. After this HF-IR, the exposed virgin male mice were mated with virgin female mice. As shown in Figure 1A, the pregnancy rate was significantly decreased when the virgin female mice were mated with irradiated male mice at 2 weeks post-IR, corresponding to the reduction of the concentration and viability of sperms in cauda epididymis of irradiated male mice (Figure 1B-C). To explore the underlying mechanism, we carried out RNA sequencing of testis tissue and further performed its functional enrichment analysis based on the database of KEGG (Kyoto Encyclopedia of Genes and Genomes). The twenty most significantly enriched signaling pathways including axon guidance pathway, Wnt signaling pathway and cAMP signaling pathway were displayed in Figure 1D, and three most relevant signaling pathways i.e. p38 MAPK, Rho GTPase and Rap1 were obtained. To demonstrate whether p38 MAPK activation played central roles in RIARE, SB202190 32 and SB203580 33, two highly specific inhibitors of p38 MAPK were administrated to male mice (12 mice per group) before HF-IR, respectively. The administration of either SB202190 or SB203580 to irradiated male mice both notably ameliorated the conception rate of mated virgin female mice from approximately 50% to nearly 80% (Figure 1E). Histopathological analysis revealed that, in comparison with the orderly and tightly arranged seminiferous tubules in sham irradiated mice, the germinal epithelium in the thoracic-irradiated mice was noticeably disrupted at 3-7 days after the last time of thoracic IR, resulting in obvious pathological changes in germ cells (Figure 1F). Specifically, dramatic interstitial oedema and mild interstitial cell infiltration was revealed at 1^st^ day post-IR (Figure 1F, arrowheads), along with irregular morphology in Sertoli cells at the 3^rd^ day after HF-IR, with the presence of vacuoles denaturation and shrinkage (Figure 1F, arrowheads). At 7^th^ day after thoracic radiation, defects in spermatid polarity was observed when spermatids no longer pointed toward the basement membrane but deviation by 90°-180° from the intended orientation (Figure 1F, arrowheads). Furthermore, exfoliation of spermatogenic cells was detected in a part of the lumens (Figure 1G). Intriguingly, it was found that SB202190 largely alleviated the disruption of seminiferous tubules in the irradiated male mice. The ratio of seminiferous tubules with interstitial cell infiltration, disorganized seminiferous epithelium or abnormal germ cells in SB202190-pretreated mice was reduced to nearly one third of the non-treated mice at 1-7 days post-IR (Figure 1G).

### P38 MAPK activation disrupts BTB-associated proteins

**Figure 2:**
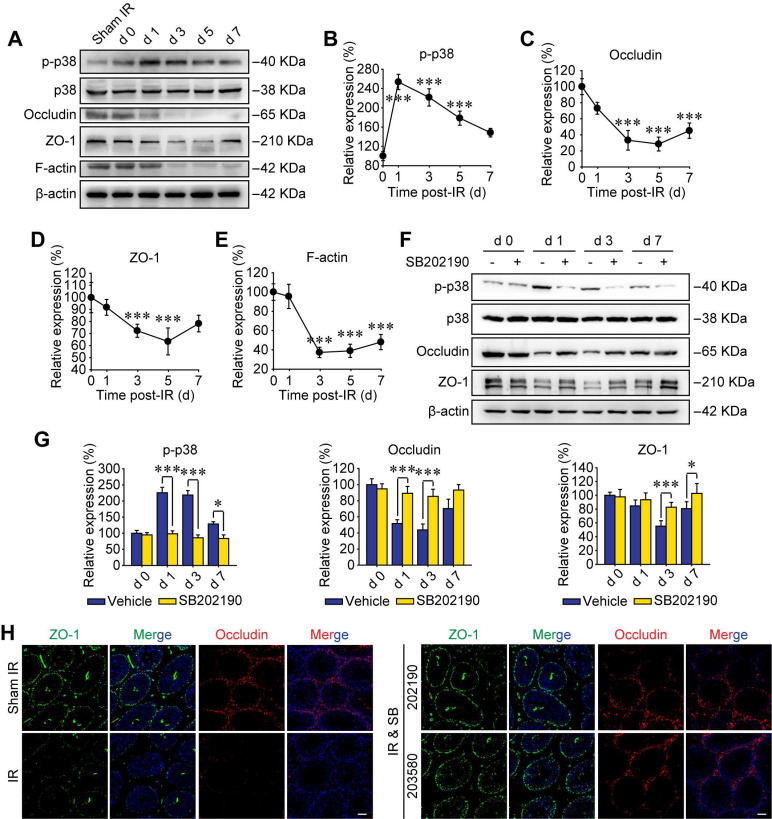
** P38 MAPK activation disrupts BTB-associated proteins. (A)** Western blot assay of the proteins in whole cell lysate of testes at the indicated time after HF-IR (8 Gy × 3) through right thorax of mice. **(B-E)** Relative expression levels (to day 0 post-IR) of p-p38, occludin, ZO-1 and F-actin after IR.** (F, G)** Western blot assay of the proteins and their relative levels in whole cell lysate of testes at the indicated time after thoracic IR with or without pre-treatment of SB202190. SB202190 was administrated to mice (25 μg/kg/d, i.p. for 3 days) 2 h ahead of HF-IR.** (H)** Representative immunofluorescence staining images illustrate the distribution of ZO-1 (green) and occludin (red) in testes during homeostasis or at 3 days after thoracic IR with or without pre-treatment of SB202190 or SB203580. Three mice for each group. (Scale bar = 50 µm).

Recently, p38 signal was proved to play key roles in paternal stress-induced telomere shortening of mouse testicular germ cells in the offspring 34, indicating the effect of p-p38 upon sperm's viability of male offspring. In addition, p38 MAPK signaling pathway was reported to participate harmful environmental pollutant (*e.g.* polystyrene microplastics)-induced sperm damage 35. However, the molecular mechanisms are not elucidated. To further verify the biological effects of p38 MAPK activation on RIARE, we underwent Western blot assay to analyze the expression of phosphorylated p38 in testicular tissue post-IR. As shown in Figure 2A, p38 MAPK was evidently phosphorylated to a high level at the first day after thoracic IR and then gradually decreased until the day 7 post-IR. Strikingly, the activation of p38 MAPK within testes was followed by a significant reduction of BTB-associated proteins of occludin, ZO-1 and F-actin from the day 3 post-IR, indicating a disruption of the BTB integrity (Figure 2B-E). As expected, SB202190 notably reversed the activation of p38 MAPK in testicular tissues after thoracic IR (Figure 2F). Meanwhile, the down-regulation of occludin and ZO-1 at 1-7 days after IR was also dramatically alleviated by the p38 MAPK inhibitor (Figure 2G). Immunofluorescence staining assay illustrated that the fluorescence of both ZO-1 and occludin was down-regulated so that the continuous fluorescence signals around seminiferous tubules were disintegrated in the testis of thoracic-irradiated mice, which could be rescued by SB202190 or SB203580 pre-treatment (Figure 2H). Accordingly, these results suggest that the BTB-associated proteins were disrupted through the activation of p38 MAPK pathway in thoracic-irradiated male mice.

### P38 MAPK inhibitor ameliorates BTB integrity by reversing Rac1 reduction

**Figure 3:**
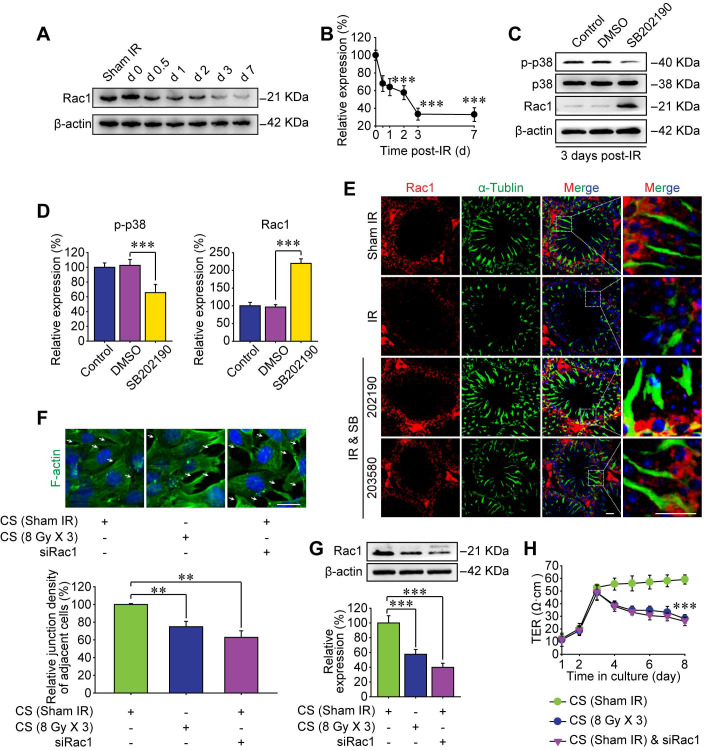
** P38 MAPK inhibitor ameliorates BTB integrity by inhibiting Rac1 downregulation. (A, B)** Western blot assay of Rac1 protein in whole cell lysate of testes and its relative level (to day 0 post-IR) at the indicated time after thoracic IR (8 Gy × 3). **(C, D)** Western blot assay of p38 and Rac1 proteins in whole cell lysate of testes and their relative protein levels at 3 days after thoracic IR with or without pre-treatment of SB202190 or its solvent control of DMSO. SB202190 was administrated to mice (25 μg/kg/d, i.p. for 3 days) 2 h ahead of HF-IR.** (E)** Representative immunofluorescence staining images illustrated the distribution of Rac1 (red) and α-tubulin (green) in testes during homeostasis or 3 days after thoracic IR (Scale bar = 25 µm).** (F)** Representative immunofluorescence staining images of TM4 Sertoli cells* in vitro*, and the relative junction density of adjacent cells was detected. Cells with or without siRac1 transfection were treated with indicated CS for 3 days. Scale bar = 10 µm. **(G)** Western blot assay of Rac1 protein in the whole cell lysate of TM4 Sertoli cells treated with CS from thoracic-irradiated mice or transfected with siRac1 for 48 h.** (H)** TER detection of primary Sertoli cells treated with siRac1 transfection and indicated CS for 5 days. ***p < 0.001.

Rho GTPase family is a critical downstream target of p38 MAPK 16, which was also enriched as the second relevant signaling pathway of RIARE in Figure 1D. The Rac subfamily of Rho GTPases includes Rac1, Rac2 and Rac3, which shares high similarity with each other 36. Rac1 is ubiquitously expressed 37, Rac2 is expressed in hematopoietic cells 38 and Rac3 mRNA is expressed mainly in the brain tissue 36. Therefore, the most well-known member of Rho GTPase family, Rac1, was examined by Western blot in this study. As shown in Figure 3A, the expression level of Rac1 protein in testes was gradually decreased along the time after HF-IR on right thorax of mice and it reduced to approximately 35% of sham control group at 3 days post-IR and still maintained at a low level till 7 days post-IR (Figure 3B). Interestingly, SB202190 pre-treatment dramatically reversed IR-induced attenuation of Rac1 by inhibiting p38 MAPK signal (Figure 3C-D). Meanwhile, given that polar cytoskeleton protein α-tubulin is reported to be co-localized with BTB-associated protein, most notably F-actin 39, we speculated that α-tubulin might be involved in Rac1-induced BTB dynamics. As shown in Figure 3E, Rac1 reduction was manifested to cause severe degradation of cytoskeleton protein α-tubulin in seminiferous tubules of thoracic irradiated mice, which was also ameliorated by p38 MAPK inhibition (Figure 3E). Of note, Rac1 mainly existed in the cytoplasm of Sertoli cells, partly co-localizing with α-tubulin, indicating that Rac1 might regulate the BTB dynamics through the interaction with cytoskeleton proteins.

To further confirm the pivotal role of Rac1 in regulating the BTB integrity, *in vitro* cellular experiments based on Rac1 siRNA (siRac1) interfering was carried out. Sertoli cells co-cultured with conditioned serum (CS) from thoracic-irradiated mice acted as a positive control. Importantly, Rac1 silencing elicited notable disruption of F-actin distribution in Sertoli cells and thus diminished the cell-to-cell connection (Figure 3F-G). TER across the cell epithelium was monitored to further indicate the connection status among Sertoli cells. As shown in Figure 3H, the TER value was reduced by the treatment of CS or siRac1 to one third of the basal level at day 1 after the treatment and further declined to approximately 50% of control group at day 5 after the treatment. The reason may be that when the adhesion between cells is relaxed, the current flowing between the opened gaps increases, indicating the enhancement of the barrier permeability of Sertoli cells treated with CS or transfected with siRac1. These results demonstrated that the BTB disruption in thoracic IR-treated mice was significantly correlated with the down-regulation of Rac1.

### Abscopal BTB disruption is driven by Rac1 nuclear translocation

**Figure 4:**
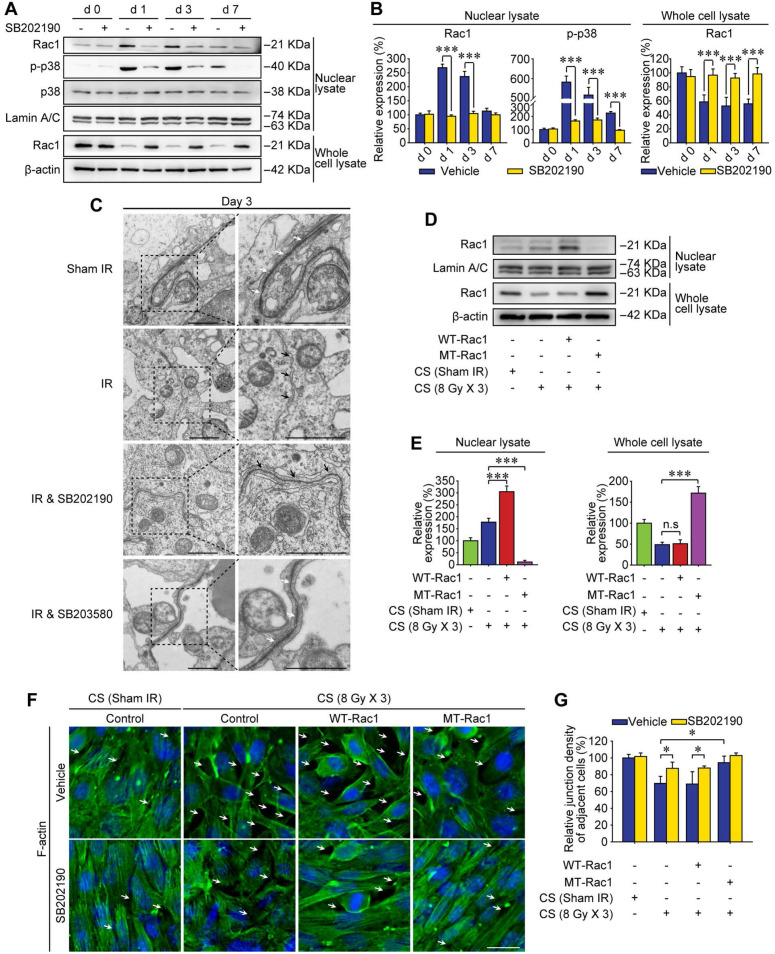
** Abscopal BTB disruption is driven by Rac1 nuclear translocation. (A)** Mice were pretreated with SB202190 (25 μg/kg/d, i.p. for 3 days) 2 h ahead of HF-IR (8 Gy × 3). Western blot assay of proteins in nuclear lysate and whole cell lysate of testes at the indicated time after thoracic IR with or without pre-treatment with MAPK inhibitor SB202190. Testis tissue from three mice for each group at indicated time. Representative data from three independent experiments were illustrated. **(B)** Relative expression levels of above measured proteins in testes after IR.** (C)** TEM analysis of the integrity of the BTB at 3 days after thoracic IR with or without pre-treatment of SB202190 (25 μg/kg/d, i.p. for 3 days) or SB203580 (50 mg/kg/d, i.p. for 3 days). Three mice for each group. Scale bar = 2 µm. **(D)** Western blot assay of Rac1 protein in nuclear lysate and whole cell lysate of WT-Rac1 or MT-Rac1 overexpressing TM4 Sertoli cells after co-cultured with the conditioned serum (CS) from thoracic irradiated mice. Cell lysates were extracted at 24 h after CS co-culture. **(E)** Relative expression level (to sham IR group) of Rac1 in Sertoli cells indicated in Figure 4D.** (F)** Representative immunofluorescence staining images of F-actin protein in TM4 Sertoli cells co-cultured with CS with or without SB202190 (10 μM) for 72 h* in vitro*. Scale bar = 10 µm. **(G)** Relative junction density of adjacent cells was detected. *p < 0.05, ***p < 0.001.

Subcellular localization of proteins is an important factor for better understanding the molecular mechanisms of the RIARE. Given that cytoplasmic Rac1 was strongly correlated with the BTB integrity in mice exposed to HF-IR, we sought to determine the subcellular localization of Rac1 post-IR. Interestingly, in concomitant with IR-induced p38 MAPK activation, Rac1 content in the nuclear lysate of testes was increased, while Rac1 in the whole cell lysate was decreased at 1-7 day after thoracic IR, indicating the nuclear translocation of Rac1 (Figure 4A-B). Moreover, HF-IR also led to ultrastructural changes in the BTB. As shown in Figure 4C, TJs that constitute the BTB (arrowheads) between two adjacent Sertoli cells in sham irradiated mice were clearly identified by electron microscopy. Yet the TJs of BTB became opened and impaired after thoracic IR. This pattern is somewhat similar to TJs in rodent testes before BTB was formed 40. As expected, both IR-induced Rac1 nuclear transport, p-p38 upregulation, and BTB disruption in testes post-IR were reversed by the p38 MAPK inhibitor treatment of mice (Figure 4A-C).

In order to identify whether Rac1 reduction in the abscopal testes was geared by Rac1 nuclear translocation, mutant (MT) Rac1 with mutation in the nuclear translocation sequence was transfected into Sertoli cells. As shown in Figure 4D-E, nuclear translocation of Rac1 in Sertoli cells treated with conditioned serum was notably enhanced by wild-type (WT)-Rac1 overexpression while abrogated by MT-Rac1 transfection. Interestingly, WT-Rac1 failed to reverse CS-induced Rac1 degradation, yet MT-Rac1 maintained the expression level of Rac1 in CS-treated Sertoli cells. Moreover, compared with WT-Rac1 overexpressing Sertoli cells whose cell-to-cell connection could be abrogated by the CS from irradiated mice, the Sertoli cells with MT-Rac1 were much more resistant to CS-induced disruption of F-actin (Figure 4F-G). The reason might be that the reduction of Rac1 in Sertoli cells was geared by p38-mediated Rac1 nuclear translocation (Figure 4A, C), since transfection of WT-Rac1, which was capable of nuclear transport, failed to reverse Rac1 protein degradation and subsequent BTB disruption. Contrarily, incapable of Rac1 translocation into nuclei, MT-Rac1 retained the cytoplasmic distribution and thus prevented BTB disruption induced by p38-drived Rac1 nuclear translocation. As expected, p38 inhibitor, SB202190, significantly relieved CS-induced disruption of F-actin in Sertoli cells (Figure 4F-G). These data revealed that Rac1 nuclear transport might be the most critical effector regulated by p38 MAPK in RIARE.

### Rac1 nuclear translocation in abscopal testes is driven by YWHAZ

**Figure 5:**
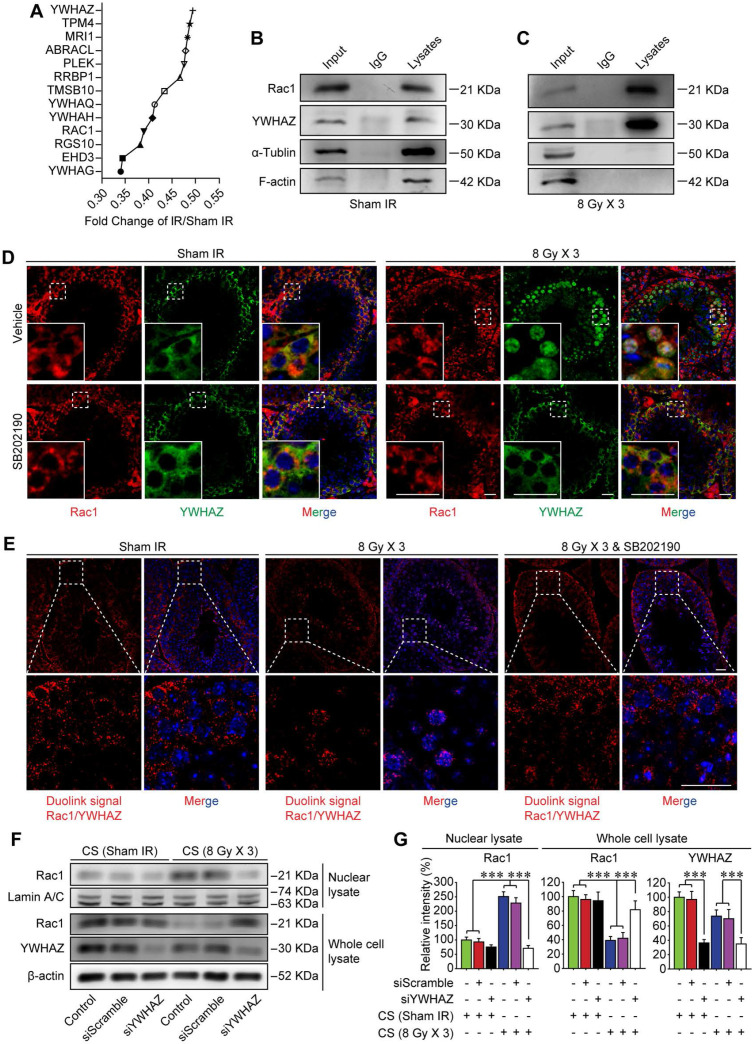
** Rac1 nuclear translocation in abscopal testes is driven by YWHAZ. (A)** Relative protein expression levels in mice serum at 3 days after thoracic IR as detected by tandem mass tag (TMT) quantitative proteomic analysis. **(B, C)** Co-IP assay assessed the protein-protein interactions between Rac1, YWHAZ, α-tubulin and F-actin during homeostasis** (B)** and 1 day after thoracic IR **(C)**. Testis lysates were subjected to co-IP assay with anti-Rac1 antibody (1 µg, ab33186, Abcam) as the precipitating antibody, and the blots were probed with antibody to YWHAZ, α-tubulin or F-actin.** (D)** Representative immunofluorescence staining images illustrate the subcellular co-distribution of Rac1 (red) and YWHAZ (green) in testes during homeostasis or 1 day after thoracic IR (Scale bar = 25 µm).** (E)** Representative images of individual immunofluorescence staining of Rac1 and YWHAZ interaction in the cytoplasm or nucleus in mouse testes by Duolink PLA. The red dots (Rac1/YWHAZ interaction) indicate their interaction. DAPI as a nuclear marker (Scale bar = 25 µm).** (F, G)** Western blot assay of Rac1 and YWHAZ proteins and their relative expression levels in the YWHAZ silencing TM4 Sertoli cells. After 24 h of YWHAZ siRNA transfection, cells were co-cultured with CS for another 24 h. ***p < 0.001.

Sertoli cell-formed BTB is polarized, which separates earlier-stage spermatogenic cells basally and later-stage cells apically within the seminiferous epithelium of adult testes 11. Recently, it was revealed that Rac1 deficiency in Sertoli cells was correlated with defects in cell polarity, which was manifested by the mis-localization of polarity proteins and the disruption of the BTB 20. While the mechanism remained elusive. Using tandem mass tag (TMT) quantitative proteomic analysis, YWHAZ, one of the most crucial polarity proteins in Sertoli cells was revealed to be down-regulated after thoracic IR (Figure 5A). Therefore, it could be speculated that YWHAZ might participate in Rac1 reduction-induced instability of the BTB after thoracic IR. To verify this assumption, the co-immunoprecipitation (co-IP) assay was performed, and YWHAZ was detected in the testicular Rac1 precipitate accompanied with cytoskeleton proteins of F-actin and α-tubulin, indicating a direct protein-protein interaction of YWHAZ, F-actin and α-tubulin with Rac1 (Figure 5B). Interestingly, the combination of testicular Rac1 with cytoskeleton proteins of F-actin and α-tubulin was significantly abrogated in the thoracic IR-treated mice (Figure 5C), correlating with the co-localization of Rac1 and YWHAZ in Sertoli cell nuclei (Figure 5D). Specifically, the immunofluorescence staining image intuitively displayed that the fluorescence of testicular Rac1 was intruded into the Sertoli cell nuclei together with YWHAZ in the testes of thoracic-irradiated mice, while they remained in the Sertoli cell cytoplasm in sham IR-treated or SB202190 pre-treated irradiated cohorts (Figure 5D). To further confirm the Rac1/YWHAZ interaction during thoracic IR-induced Rac1 nuclear translocation, Duolink PLA was performed using anti-Rac1 and anti-YWHAZ antibodies. Duolink PLA allows for endogenous detection of protein interactions in fixed cells and tissue samples 29. As shown in Figure 5E, the interaction (red dots) between Rac1 and YWHAZ was translocated into nuclei in HF-IR-treated mouse testis, while remained cytoplasmic in SB202190-treated cohorts, indicating a protein-protein co-localization between Rac1 and YWHAZ from cytoplasm (pre-IR) to nucleus (post-IR). In order to determine whether YWHAZ was indispensable in mediating Rac1 nuclear translocation, we knocked-down YWHAZ by siRNA in Sertoli cells and then treated the cells with CS of thoracic irradiated mice. As shown in Figure 5F-G, nuclear translocation of Rac1 in the CS-treated Sertoli cells was apparently abolished by YWHAZ abrogation, suggesting the YWHAZ-mediated Rac1 nuclear translocation during thoracic HF-IR. Taken together, these findings demonstrated that the polarity protein YWHAZ was an indispensable mediator in HF-IR-induced nuclear translocation of testicular Rac1 and subsequent BTB disruption.

### TNF-α drives testicular Rac1 nuclear translocation and RIARE

**Figure 6:**
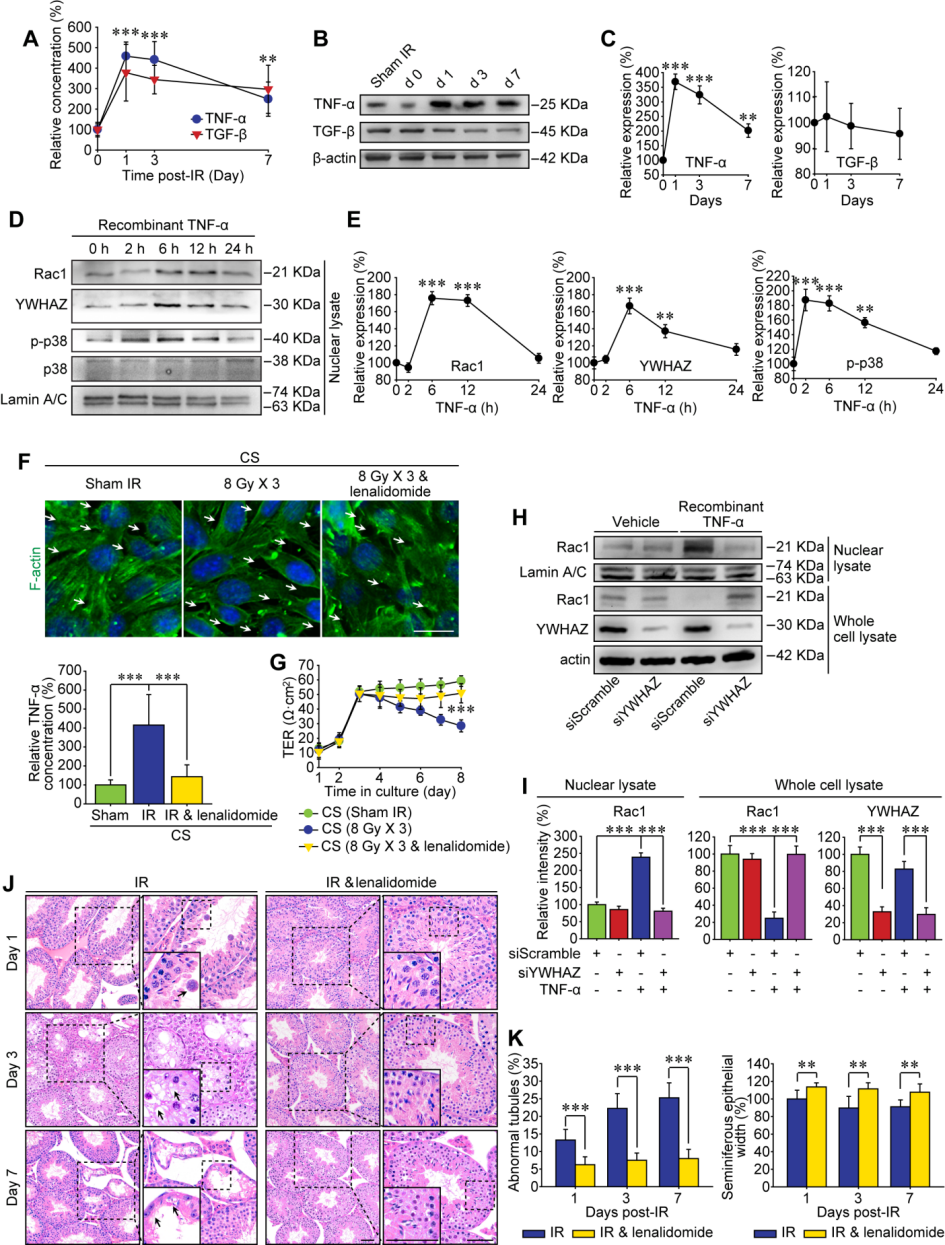
** TNF-α drives testicular Rac1 nuclear translocation and RIARE. (A)** ELISA assay of TNF-α and TGF-β concentration in mice serum at 1-7 days after thoracic IR.** (B, C)** Western blot assay of TNF-α and TGF-β in the testes at the indicated time after thoracic IR, and their relative expression levels. **(D, E)** Western blot assay of the nuclear lysate of TM4 Sertoli cells treated with recombinant TNF-α (1.0 ng/ml) for the indicated time, and their relative expression levels. **(F)** Representative immunofluorescence staining images of TM4 Sertoli cells* in vitro*. Cells were treated with CS from thoracic irradiated mice pretreated with or without TNF-α inhibitor lenalidomide (50 mg/kg/d, i.p. for 3 days) for 3 days. Scale bar = 10 µm. **(G)** TER detection of primary Sertoli cells treated with indicated CS for 5 days. **(H, I)** Western blot assay of the Rac1 and YWHAZ in the lysates of YWHAZ silencing TM4 Sertoli cells and their relative expression levels. After 24 h of YWHAZ siRNA transfection, cells were co-cultured with recombinant TNF-α (1.0 ng/ml) for another 24 h. **(J)** Representative images of HE staining of seminiferous tubules after thoracic IR with or without lenalidomide treatment (n=8). Lenalidomide was pretreated (50 mg/kg/d, i.p. for 3 days) 2 h ahead of HF-IR. Scale bars = 20 µm.** (K)** Defects in spermatogenesis in indicated groups were scored using n=8 mice for histological analysis as noted in (J). **p < 0.01, ***p < 0.001.

The BTB reconstructs periodically under physiological condition to facilitate spermiogenesis, which is mainly promoted by endogenous cytokines such as TNF-α and TGF-β 41, 42. On the other hand, high-dose IR can induce immunogenic cell death in tumor and normal tissues which further releases damage signals known as damage-associated molecular pattern molecules (DAMPs) into the systemic circulation, thus modulating inflammatory cytokines either directly or indirectly 43. In the present study, the enzyme-linked immunoassay proved that both TNF-α and TGF-β were increased sharply in the peripheral blood of mice suffering from thoracic IR (Figure 6A), and they tended to decline at 7 days post-IR. Nonetheless, only TNF-α signal was up-regulated in testicular cells, while TGF-β had no significant changes at 1-7 day after thoracic IR (Figure 6B-C). In consequence, it could be speculated that TNF-α might drive the RIARE and YWHAZ-mediated Rac1 nuclear translocation.

To testify above assumption, we treated Sertoli cells* in vitro* with recombinant TNF-α and found that p38 MAPK was activated at 2 h after the treatment, along with subsequent nuclear translocation of Rac1 and YWHAZ from 6 h after TNF-α treatment (Figure 6D-E). To verify whether TNF-α was indispensable for Rac1-mediated cytoskeleton degradation, Sertoli cells were cultured with the CS from thoracic irradiated mice with or without pre-treatment of specific TNF-α inhibitor, lenalidomide. As shown in Figure 6F, the lenalidomide pre-treatment attenuated the concentration of TNF-α in the serum of irradiated mice, and notably ameliorated both CS-induced degradation of F-actin in Sertoli cells and CS-induced enlargement of the extracellular gaps between Sertoli cells. Consequently, the lenalidomide pre-treatment partly recovered the TER value of CS-treated cells (Figure 6G). Western blot assay further confirmed that TNF-α resulted in approximately 2.5 fold increase of Rac1 nuclear translocation in Sertoli cells in comparison with control group (Figure 6H-I), indicating TNF-α was a critical original factor of the abscopal damage in the testes of thoracic-irradiated mice. As expected, the nuclear transport of Rac1 evoked by TNF-α was evidently abrogated by siYWHAZ transfection, further confirming the indispensability of YWHAZ in modulating the sub-cellular localization of Sertoli Rac1. To demonstrate whether TNF-α abolishment could alleviate IR-induced abscopal BTB disruption, lenalidomide was administrated to mice once a day on 3 consecutive days and 2 h ahead of HF-IR as well. Histopathological assay revealed that thoracic-IR induced disruption of seminiferous epithelium was relieved by this TNF-α abrogation, as manifested by the marked improvement of germline epithelium of seminiferous tubules and less exfoliation of spermatogenic cells (Figure 6J-K).

## Discussion

The combination of radiotherapy with immunotherapy has become a fundamental revolution for radiation oncology, as the aim of which is progressing from direct tumor elimination to the modulation of tumor immune microenvironment 44. In concomitant with tumor local sterilization, hypofractionated radiotherapy always induces immunogenic cell death and releases inflammatory cytokines to elicit cytotoxic T lymphocytes and eliminate tumor oligometastasis, known as anti-tumor abscopal effect 45, 46. Our previous studies have demonstrated that some signaling factors including nitro oxide and TGF-β1 are involved in tumor cell irradiation-induced bystander effects *in vitro* 47-49. Nonetheless, the present study revealed that the hypofractionated thoracic IR could induce activation of the TNF-α/p38 axis and the subsequent abscopal damage in mice testes, resulting in decrease of reproductive capacity (Figure 1).

TNF-α has been validated as a key mediator of inflammation for the success of anti-TNF-α treatment in rheumatoid arthritis, Crohn's Disease and other related chronic inflammatory conditions 50. In the mouse testis, TNF-α is produced by pachytene spermatocytes and round spermatids, as well as by the activated monocytes and macrophages in the interstitium 51. However, the receptors of tumor necrosis factors are mainly expressed on Sertoli cells 52. By lowering the steady state of BTB-associated proteins in Sertoli cells, TNF-α is able to induce BTB restructuring rapidly and to regulate the homeostasis of spermatogensis dynamically. In the rodent testis, TNF-α has pleiotropic roles. At low levels, it acts as a survival factor, but at high concentrations, it triggers cell death 53. In the present study, we found that the inflammatory cytokine TNF-α in mice serum was evidently increased from 1 day after the last time of thoracic HF-IR and further drove RIARE (Figure 6). However, thoracic IR-induced adverse effects in other abscopal tissues such as small intestines and colons were hardly observed (data not shown), indicating that the spermatogenesis in seminiferous epithelium might be more vulnerable to IR-induced TNF-α.

Actually, in this study, the RIARE was mainly attributed to the disruption of BTB of adjacent Sertoli cells (Figure 2H and 4C). Instead of other blood-tissue barriers such as blood-brain barrier (BBB) and blood-retina barrier (BRB) that are exclusively constituted by TJs of adjacent endothelial cells of micro vessels, BTB in the mammalian testes comprises the co-existing TJs, basal ES, gap junctions and desmosome 54. The most prominently distinctive ultrastructures of BTB are the tightly packed actin microfilament bundles that are vertically sandwiched between endoplasmic reticulum and apposing cell membrane of Sertoli cells, known as the basal ES 54, 55. Herein, the F-actin that constitutes microfilament bundles in Sertoli cells was revealed to be highly susceptible to thoracic IR-induced TNF-α/p38 MAPK activation both *in vivo* and *in vitro* (Figure 2A, 3G and 6F). In concomitant with F-actin degradation, tight junction-associated proteins, *i.e.,* occludin and ZO-1 were also down-regulated from 3 days post-IR (Figure 2A and H). The spermatogenesis process is precisely safeguarded at the apical compartment behind BTB, making post-meiotic spermatid develop in an immune-privileged site in the epithelium 54. Consequently, due to the BTB disruption, the pathological meiosis and spermiogenesis rendered an increase of germ cell apoptosis followed by the decline in sperm cell number and sperm viability approximately 2 weeks after thoracic IR (Figure 1B-C). That was the time required for the spermatogenic differentiation from preleptotene spermatocytes to mature sperm cells 11. Interestingly, the RIARE seemed to be reversible, which was correlated with the rebound of the sperm cell quality (Figure 1). The reason might be the reduction of TNF-α signal (Figure 6) and recovery of BTB-associated protein expression (Figure 2A) at 7 days post-IR.

The Rac subfamily of Rho GTPases includes Rac1, Rac2 and Rac3 which share high sequence similarity (80%) 56, 57. Within the Rho GTPases family, Rac1 has been most extensively characterized 18. Recently, Rac1 has been demonstrated to be required for Sertoli cell polarity and BTB integrity during later stages of spermatogenesis 20. Although Rac1 is dispensable for the establishment and maintenance of undifferentiated spermatogonia and for fetal testis differentiation, Sertoli Rac1 in adult testis is required for later stages of spermatogenesis and spermiogenesis. Mechanistically, Rac1 abrogation results in significant mislocalization of apical proteins which are responsible for BTB structure and apico-basal polarity. The disruption of the apical polarity complex proteins, along with the disperse localization of F-actin further disrupts the spermatogenesis in adult testis. Protein nuclear translocation plays critical roles in biological progressions, such as signaling transduction, cell adhesion and regulation of cell polarity 58, 59. In the present study, Sertoli Rac1 was found to translocate into nucleus due to thoracic IR-induced p38 MAPK activation (Figure 4). Moreover, using co-IP assay, immunofluorescence analysis and Duolink PLA, it was demonstrated that Rac1 was closely co-localized with YWHAZ, a critical polarity protein in testicular Sertoli cells (Figure 5B-E). Of note, the interaction between Rac1 and YWHAZ was demonstrated to be indispensable for Rac1 nuclear translocation (Figure 5F-G).

Previous work has demonstrated that the 14-3-3 family regulates the transport of their ligands between nuclei and cytoplasm 60, indicating a pivotal role of 14-3-3 in regulating signaling transduction, as a polarity protein. Moreover, 14-3-3-mediated cellular sublocalization is dependent on nuclear localization signals 60. C-terminal polybasic region of Rac1 comprises a significant functional nuclear localization signal, which is crucial for Rac1 nuclear transport 61-63. Therefore, this study applied the mutated nuclear translocation sequence of Rac1 to further investigate the molecular mechanisms of TNF-α/p38/Rac1 axis in regulating RIARE. As expected, YWHAZ-mediated nuclear translocation of Rac1 was highly dependent on the specific nuclear translocation sequence of Rac1 (Figure 4D-E). Intriguingly, p38 MAPK signal-induced BTB disruption was evidently ameliorated by MT-Rac1 transfection (Figure 4F-G), suggesting the critical role of cytoplasmic Rac1 played in stabilizing BTB structure. However, WT-Rac1 overexpression failed to rescue p38 MAPK-elicited BTB disruption.

Interestingly, IR-induced Rac1 nuclear transport further led to loss of BTB-associated proteins and Rac1 itself (Figure 3) and even YWHAZ (Figure 5). The reason might be that the cytoplasmic combination of Rac1 and cytoskeleton proteins such as α-tubulin is pivotal for stabilization of individual protein and BTB. However, Rac1 nuclear translocation disconnects the combination of polarity proteins with BTB-associated cytoskeletons and thus causes destabilization of BTB. This mechanism was further confirmed by WT-Rac1 overexpression in Sertoli cells, given that even WT-Rac1 (capable of nuclear translocation) overexpression failed to reverse the reduction of Sertoli Rac1 driven by p38-mediated Rac1 nuclear translocation (Figure 4A and 4D).

**Figure 7:**
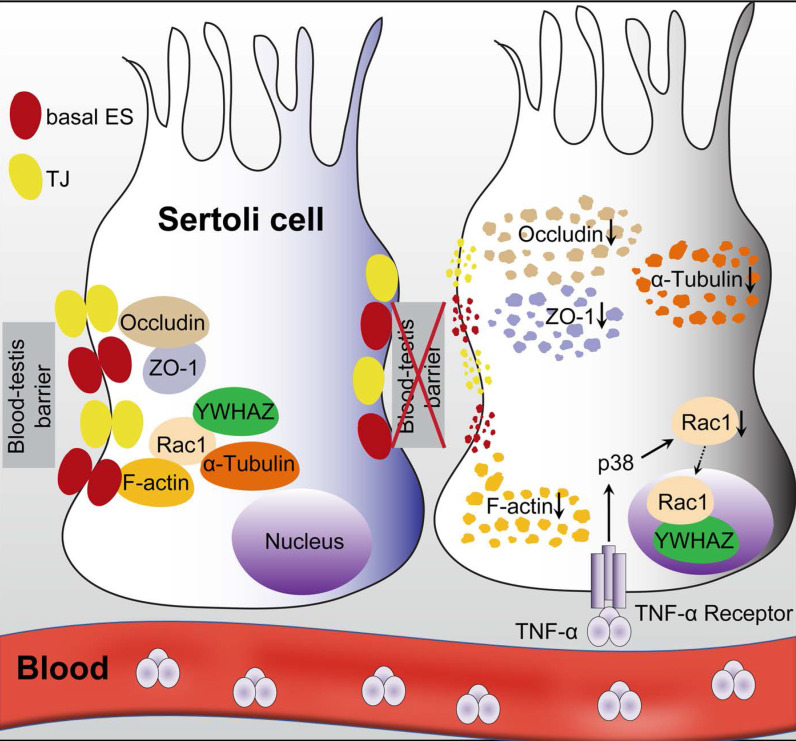
** A proposed model illustrating IR-induced abscopal BTB disruption driven by Sertoli Rac1 nuclear translocation.** Polarity protein of Rac1 was mainly localized in the cytoplasm together with cytoskeletons (α-tubulin) and BTB-associated proteins such as F-actin during homeostasis, which maintained the polarity of Sertoli cells and the integrity of the basal ectoplasmic specialization (ES) and TJs (left). Nonetheless, thoracic HF-IR induced activation of TNFα/p38 MAPK signal in abscopal Sertoli cells, which led to nuclear transport of Rac1 in an YWHAZ dependent manner. Abrogation of the protein-protein interaction of Rac1 with cytoskeletons (α-tubulin) and BTB-associated proteins (F-actin) made these proteins more susceptible to degradation, thus disrupted the cell polarity and BTB integrity in the abscopal Sertoli cells (right).

Overall, the present study clearly illustrated that Sertoli cell-constituted BTB appeared to be the target of RIARE, and YWHAZ-mediated Sertoli Rac1 nuclear translocation was pivotal to TNFα/p38 MAPK-induced reproductive dysfunction in thoracic-irradiated male mice (Figure 7). Mechanistically, Rac1 was mainly localized in the cytoplasm together with cytoskeletons and BTB-associated proteins during homeostasis, which maintained the polarity of Sertoli cells. During thoracic HF-IR, TNFα/p38 MAPK-induced Rac1 nuclear translocation abrogated the protein-protein interaction between Rac1 and BTB-associated proteins, which made these proteins more susceptible to degradation, and thus disrupted the BTB integrity in the abscopal Sertoli cells. Therefore, targeting the TNFα/p38 MAPK/Rac1 axis may be promising strategies for alleviating RIARE in young male patients receiving hypofractionated radiotherapy.

## Abbreviations

BBB: blood-brain barrier
BRB: blood-retina barrier
BTB: blood-testis barrier
Co-IP: co-immunoprecipitation
CS: conditioned serum
DAMP: damage-associated molecular pattern molecule
DAPI: 4′,6-diamidino-2-phenylindole
DEGs: differentially expressed genes
DMSO: dimethyl sulfoxide
Elisa: enzyme-linked immunoassay
ES: ectoplasmic specialization
FC: fold change
HE: hematoxylin and eosin
HF-IR: hypofractionated irradiation
IR: irradiation
KEGG: Kyoto Encyclopedia of Genes and Genomes
MAPK: mitogen activated protein kinase
MT: mutant
mTORC1: rapamycin kinase complex 1
PBS: phosphate-buffered physiological saline
PFA: paraformaldehyde
RIARE: radiation-induced abscopal reproductive effect
SCs: Sertoli cells
SRA: Sequence Read Archive
siRac1: Rac1 siRNA
TEM: transmission electron microscopy
TER: transepithelial electrical resistance
TGF-β: transforming growth factor-β
TJ: tight junction
TMT: Tandem mass tag
TNF-α: tumor necrosis factor-α
WT: wild-type
ZO-1: zonula occludens-1
